# Supplementary material for: Grapevine Leafroll-Associated Virus 3 in Single and Mixed Infections Triggers Changes in the Oxidative Balance of Four Grapevine Varieties
Source: Int J Mol Sci. 2022 Dec 20;24(1):8. doi: 10.3390/ijms24010008 (PMC9819915; doi:10.3390/ijms24010008)
Supplement: Supplementary file 1 [file ijms-24-00008-s001.zip › Supporting info_Hancevic et al.pdf]

Table S1. Abbreviations: Physiological parameters as indicators of host-plant response to viral infection

No.of parameters	Symbols	Name of parameter	Type of parameter
1	**P**	Phosphorus	**Macro and micro nutrients**
2	**K**	Pottasium	
3	**N**	Nitrogen	
4	**Ca**	Calcium	
5	**Zn**	Zinc	
6	**Mn**	Manganese	
7	**Fe**	Iron	
8	**Mg**	Magnesium	
9	**Cu***	Copper	
10	**SOD**	Superoxide dismutase	**Stress response**
11	**MDA**	Malondialdehyde	
12	**perox**	Hydrogen peroxide	
13	**cla**	Chlorophyl a	
14	**clb**	Chlorophyl b	
15	**car**	Carotenoids	
16	**prot**	Soluble proteins	
17	**SA***	Salicid acid	
18	**prolin**	Prolin	
19	**len_inter**	Lenght internode	
20	**DW_leaves**	Dried weight of leaves	
21	**avg_L**	Colorimetric parameters	
22	**avg_A**		
23	**avg_APSa**		
24	**avg_b**		
25	**avg_c**		
26	**avg_h**		
27	**photo (A)**	Net CO_2 assimilation	**Primary metabolism**
28	**cond (gs)**	Stomatal condundance	
29	**Ci**	Substomal CO_2 concentration	
30	**A/Ci**	A/Ci	
31	**A/gs**	A/gs	
32	**phiCO2**	Spectral CO_2 assimilation	
33	**TrmolE**	Leaf transpiration	
34	**RH_R**	Hidraulic resistance	
35	**RWC**	Relative water content	
36	**perm_memb**	Membrane permeability	

*Parameters that were measured only in one year. Cu was measured in 2021, and SA in 2020.

Table S2. Raw data of every treatment following first year of experiment (2020). Table S2 is presented in the separate document.

Table S3. Raw data of every treatment following second year of experiment (2021). Table S3 is presented in the separate document.

a

b

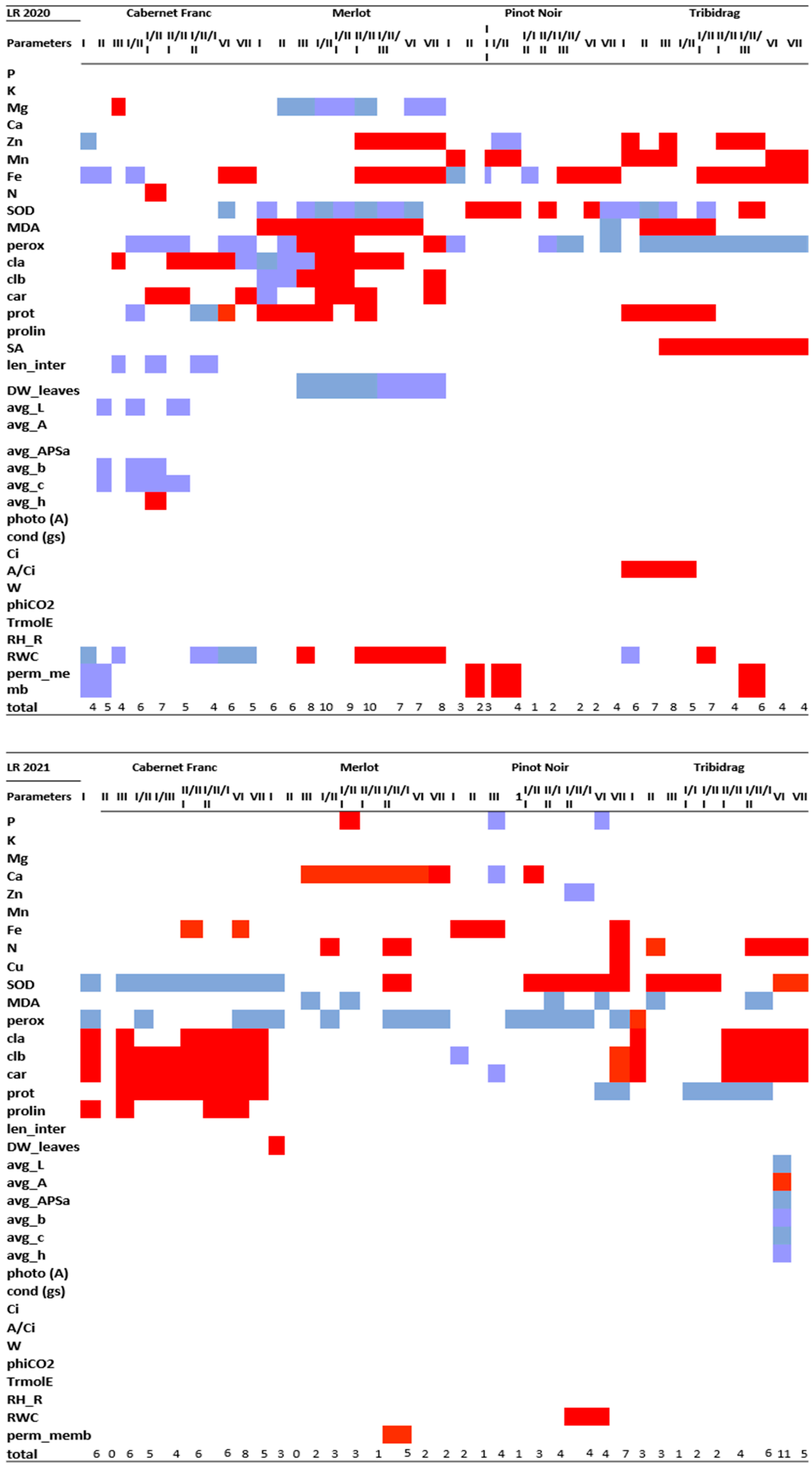

LR 2020
Cabernet Franc
Merlot
Pinot Noir
Tribidrag
Parameters
P
K
Mg
Ca
Zn
Mn
Fe
N
SOD
MDA
perox
cla
clb
car
prot
prolin
SA
len_inter
DW_leaves
avg_L
avg_A
avg_APSa
avg_b
avg_c
avg_h
photo (A)
cond (gs)
Ci
A/Ci
W
phiCO2
TrmolE
RH_R
RWC
perm_me
mb
total
LR 2021
Cabernet Franc
Merlot
Pinot Noir
Tribidrag
Parameters
P
K
Mg
Ca
Zn
Mn
Fe
N
Cu
SOD
MDA
perox
cla
clb
car
prot
prolin
len_inter
DW_leaves
avg_L
avg_A
avg_APSa
avg_b
avg_c
avg_h
photo (A)
cond (gs)
Ci
A/Ci
W
phiCO2
TrmolE
RH_R
RWC
perm_memb
total

Figure S1. Genotype effect for leafroll (LR) isolates (I, II, III, I/II, I/III, II/III, I/II/III, VI and VII) in 2020 (a) and in 2021 (b) in relation to control plants. Significant changed are presented as colored squares according to t-test (*P* < 0.05; red-value decreases; blue-value increases).

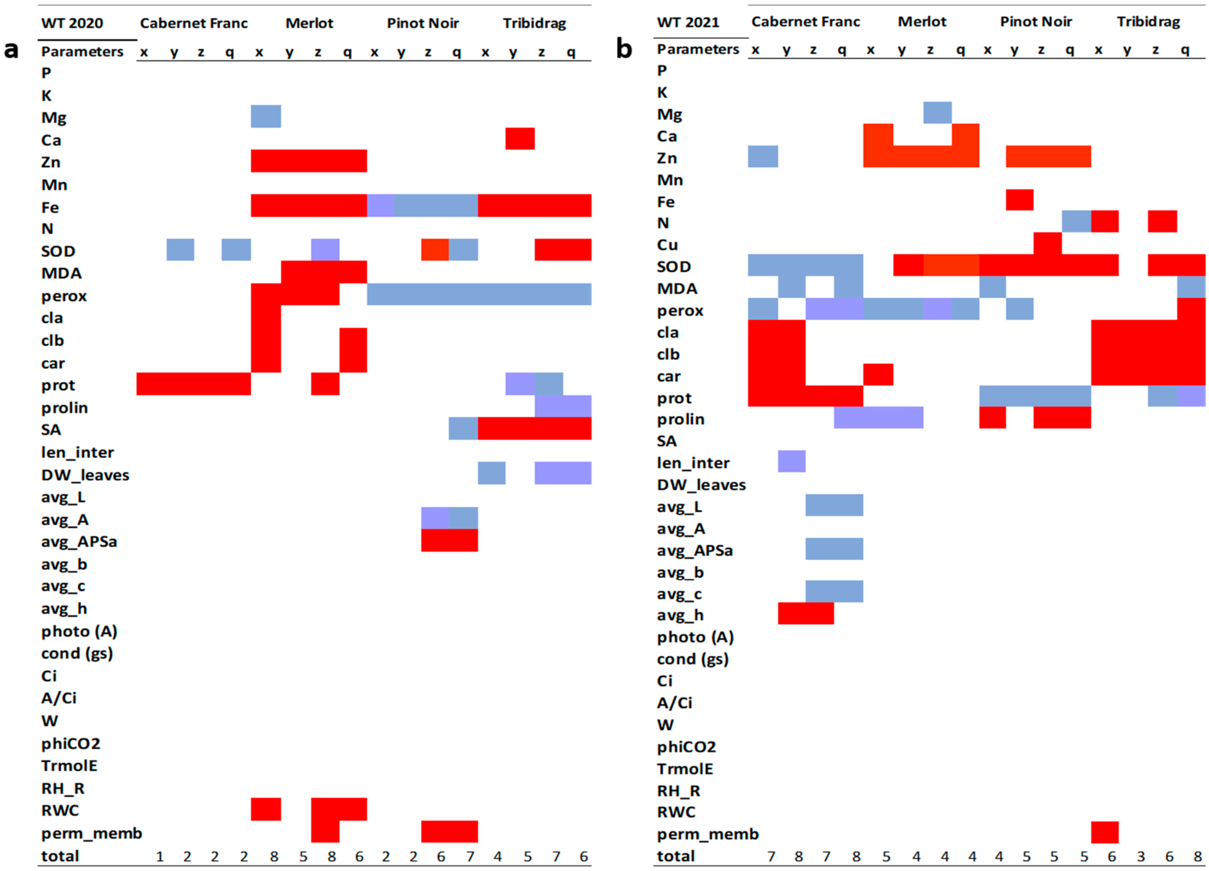

Figure S2. Genotype effect for wild-type (WT) isolates (X, Y,Z and Q) in 2020 (a) and in 2021 (b) in relation to control plants. Significant changed are presented as colored squares according to t-test (*P* < 0.05; red-value decreases; blue-value increases).

Table S4. Intensity of leafroll symptoms observed in Cabernet Franc, Merlot, Pinot Noir and Tribidrag variety infected with leafroll (LR) genotypes (I, II, III, I/II, I/III, II/III, I/II/III, VI and VII) and wild-type (WT) genotypes (X, Y, Q and Z) in the season 2021.

Tretmans	Repetitions	Cabernet Franc	Merlot	Pinot Noir	Tribidrag
Control	1				
	2				
	3				
	4				
	5				
I	1	*	***		
	2	*	**		
	3	**	**		
	4	***	**		
	5	***	*	*	
II	1	*	*		
	2		*	**	

	3		***	*	
	4		**	*	
	5	*	**	*	
**III**	1	*		**	
	2	*		**	
	3	**		**	
	4			**	
	5	**	*	**	
**I/II**	1	***			
	2	**	*	*	
	3	**	*		
	4	*	*	*	
	5	**	*	*	
**I/III**	1	***	***		
	2	***			
	3	***			
	4	*			
	5	*	*	*	
**II/III**	1	**	*		
	2	*	*		
	3	*	*		
	4	**	*	*	
	5	*	*	*	
**I/II/III**	1		*		
	2	*	*		
	3	***	**		
	4	**	*		
	5	*	*		
**VI**	1				
	2				
	3	*	**	*	**
	4			*	*
	5			*	
**VII**	1			*	
	2				
	3			*	
	4	*	***	*	
	5	**		*	
**X**	1		**	***	
	2		*	***	**
	3			***	*
	4		**	***	*
	5	**	**	*	
**Y**	1	*	*	***	*
	2	*	*	***	**
	3	**	*		*
	4	**	*	**	*
	5	***	**	***	*
**Q**	1	***	*		
	2	**	***		
	3	**			*
	4	*			
	5	***	*		
**Z**	1		*	*	**
	2	**	***	*	*
	3	*	**	***	
	4	**	*		
	5	**	**		

Symptoms were ranked as asymptomatic ( ), mild (*), moderate (**) and severe (***).
